# Editorial: Antimalarial chemotherapy in the XXIst century

**DOI:** 10.3389/fphar.2022.1118683

**Published:** 2022-12-20

**Authors:** Paula Gomes, Rafael V. C. Guido

**Affiliations:** ^1^ LAQV-REQUIMTE, Department of Chemistry and Biochemistry, Faculty of Sciences (FCUP), University of Porto, Porto, Portugal; ^2^ São Carlos Institute of Physics, University of São Paulo, São Carlos, Brazil

**Keywords:** plasmodium, inhibitor, drug discovery, resistance, malaria

*Plasmodium* parasites are known as the etiological agents of malaria, transmitted by *Anopheles* mosquitoes. Malaria is one of the “Big Three” infectious diseases, alongside HIV-1/AIDS and Tuberculosis, that are prioritized by the World Health Organization (Bourzac, 2014). While tackling the “Big Three” is an important part of the Sustainable Development Goal 3—Good Health and Wellbeing—of the United Nations Agenda for 2030, the emergence of the COVID-19 pandemic reversed decades of improvements and led to a notable increase in malaria deaths in Sub-Saharan Africa in 2020 (World Health Organization, 2021). Therefore, malaria remains a major health concern worldwide. This accentuates the pressing need to control malaria. The use of insecticide-treated bed nets and artemisinin-based combination therapies (ACTs) have been major strategies in decreasing the malaria burden over the past 2 decades (World Health Organization, 2021). Nevertheless, the emergence of parasite resistance to ACT, now also spread to Africa, is a serious menace to malaria containment and drives the need to find efficient alternatives (Ashley et al., 2014; Uwimana et al., 2020; Balikagala et al., 2021; Noreen et al., 2021; Rosenthal, 2021). This scenario has fueled new approaches towards antimalarial drug discovery and development, causing an impressive enrichment of the antimalarial leads’ portfolio in the XXIst century (Burrows et al., 2011; Burrows et al., 2017; Aguiar et al., 2019).

An optimal antimalarial should not elicit resistance and should neutralize all parasite stages inside the human host, namely 1) hepatic forms associated with the asymptomatic onset of disease, 2) dormant liver forms (hypnozoites) responsible for relapse in certain malaria types, 3) blood-stage forms underlying the symptomatic phase of infection, and 4) gametocytes, responsible for host-to-vector transmission. An ideal antimalarial should additionally be produced at a low cost since malaria is mainly endemic in low-to-middle-income countries (Burrows et al., 2017). Besides conventional drug discovery approaches, cost-effective ways to accelerate the identification of novel antimalarial strategies have included 1) combination therapy, by joining two or more known drugs (van der Pluijm et al., 2021), 2) covalent bitherapy, by conjugating different pharmacophores (Odhiambo et al., 2017), 3) repurposing of drugs with other therapeutic indications (Pazhayam et al., 2019), and 4) rescuing of known antimalarial scaffolds (Silva et al., 2020). This delivered a significant Research Topic of new antimalarial candidates, several of which showed potential to step into clinical trials. Yet, the enthusiasm for progressing such candidates has been cooling down and, except for tafenoquine (Campo et al., 2015), no new molecule has been approved for clinical use against malaria since the introduction of ACT at the dawn of this century. Provided the current scenario, this Research Topic conveys a broad picture of the current challenges and prospects in antimalarial drug discovery and development.

The Research Topic “Antimalarial Chemotherapy in the XXIst Century” offers six articles, including three literature reviews and three original research articles addressing the scope and limitations of antimalarial drug discovery and development. Gil and Fançony reviewed the capacity of the lethal form of the parasite, *Plasmodium falciparum*, to develop resistance against antimalarial drugs. In this sense, they described the pivotal role of drug transporters in the capacity of the parasite to evade drug action. They focused on the association of Multidrug Resistance Proteins (MRPs) with *in vivo-* and *in vitro*-altered drug responses, and how MRPs are central factors for developing multi-drug resistance phenotypes. Monteiro Júnior et al. reviewed the role of transport proteins, as well. The authors argued that the transporters-mediated solutes’ uptake is essential for intracellular parasite proliferation and survival. Therefore, investigating transporters as molecular targets for antimalarial drug discovery can be an attractive strategy to eliminate the parasite. The third review article on the Research Topic focused on artemisinin resistance and malaria elimination. Hanboonkunupakarn et al. described the pharmacological properties of ACTs. In addition, they reviewed the molecular mechanisms of artemisinin resistance and the potential changes needed in the treatment regimens to overcome this obstacle.

The Research Topic of original research articles includes both basic and applied science toward a better understanding of the biology of the malaria parasite and the discovery of new chemical tools to control the disease. In this sense, the investigation carried out by Silva et al. studied blood samples of Tanzanian patients with uncomplicated *P. falciparum* malaria infections who underwent treatment with the ACT artemether-lumefantrine. Their findings indicated that the transcription levels of pfmdr1 and pfcrt (two transmembrane transporters associated with sensitivity to several antimalarials) showed significant co-expression patterns *in vivo*, which were generally maintained during the ACT treatment. Moreover, the work highlighted the need to link valuable *in vitro* data on the parasite drug response mechanisms to the *in vivo* therapy context.

The applied original research articles reported the discovery of natural compound analogs as attractive molecular scaffolds for antimalarial development. Uddin et al. used a target-based virtual screening approach on a subset of natural compounds from the ZINC database to identify inhibitors of the parasitic cysteine proteases falcipain-2 and falcipain-3 (FP2 and FP3). Enzymatic and biophysical assays verified the inhibition and binding properties of the identified hits, which showed inhibitory activities in the low micromolar range against chloroquine-sensitive and chloroquine-resistant strains of *P. falciparum*. Furthermore, the most promising hit did not show hemolytic or cytotoxic activities against human HepG2 cells and exhibited improved antiplasmodial activity *in vivo* when administered in combination with chloroquine in a mice model infected with *P. berghei*. Abacha et al. used the ethnopharmacological knowledge of the West Africans to investigate the antiparasitic and anticancer properties of cryptolepine, an alkaloid found in *Cryptolepis sanguinolenta*. They isolated the alkaloid and applied semi-synthetic strategies to discover new analogs with improved inhibitory activities against *P. falciparum*, *P. knowlesi*, *Trypanosoma brucei*, and ovarian cancer cells. Their findings suggested that *C. sanguinolenta* can be a sustainable source of compounds that may lead to the discovery of new drug candidates for malaria, African trypanosomiasis, and cancer.

The Research Topic presents advances in the field of *P. falciparum* biology and therapy. The Research Topic includes articles describing from the molecular mechanisms underlying the emergence of resistant parasites to the application of computational and experimental approaches to discover innovative agents for malaria control. In sum, the articles on the Research Topic highlight the challenges, limitations, and future perspectives in antimalarial chemotherapy in the XXIst century.
